# The environmental impact of multi-specialty robotic-assisted surgery: a waste audit analysis

**DOI:** 10.1007/s11701-025-02278-5

**Published:** 2025-03-11

**Authors:** Gerald Tjahyadi, Patrick-Julien Treacy, Kate Alexander, Jacob Bird, Sascha Karunaratne, Scott Leslie, Kate McBride, Daniel Steffens, Ruban Thanigasalam

**Affiliations:** 1 Surgical Outcomes Research Centre (SOuRCe), Royal Prince Alfred Hospital, PO Box M157, Camperdown, Sydney, NSW 2050 Australia; 2 Faculty of Medicine and Health, Concord Clinical School, The University of Sydney, Sydney, NSW Australia; 3 Institute of Academic Surgery (IAS), Royal Prince Alfred Hospital, Sydney, NSW Australia; 4 Faculty of Medicine and Health, Central Clinical School, The University of Sydney, Sydney, NSW Australia; 5 Department of Urology, Royal Prince Alfred Hospital, Sydney, NSW Australia; 6 Department of Urology, Concord Repatriation General Hospital, Sydney, NSW Australia

**Keywords:** Robotic surgery, Robot-assisted surgery, Environment, Environmental waste, Waste audit

## Abstract

Modern advances in medicine and technology have led to an increase in robotic-assisted surgery (RAS) cases. However, concerns have been raised about the potential environmental impact of RAS. Despite this, only limited quantitative evidence is currently available. Therefore, this study aims to objectively quantify the environmental impact of a multi-specialty RAS caseload at an Australian public tertiary hospital. An analysis was performed to quantify the amount of waste produced on a consecutive multi-specialty RAS caseload performed between August 2016 and March 2023 at a major public hospital. The weight of each instrument and consumable was measured and the total weight of discarded waste was calculated for each RAS case. A total of 671 patients underwent RAS using the *da Vinci Xi* System, including Urology (*n* = 341, 50.8%), Cardiothoracic (*n* = 158, 23.5%), Gynaecology (*n* = 107, 16.0%), and Colorectal (*n* = 65, 9.7%). Overall, the amount of waste was 2,948.9 kg, with single-use items contributing the largest volume (93.9%). Cardiothoracic generated the lowest average weight of waste per case (3.04 kg) while Colorectal generated the highest (5.27 kg). Overall, the *da Vinci Xi* instrument arm drape was the single item with the largest contribution to the total weight of waste (32.9%). RAS generates substantial waste, primarily from single-use items. These findings underscore the need for strategies to mitigate the environmental footprint of RAS as its adoption continues to grow.

**Supplementary Information:**

The online version contains supplementary material available at 10.1007/s11701-025-02278-5.

## Introduction

Modern advances in medicine and technology in recent years have led to an increase in the uptake of robotic-assisted surgery (RAS), which has emerged as the next frontier of minimally invasive surgery. RAS is linked with improved clinical outcomes in colorectal or prostate cancer surgeries, including shorter hospital stays, better post-operative function, less blood loss, and reduced post-operative complications [1–3]. Each new iteration of a robotic surgical device has endeavoured to become more effective and safer, building upon previously established systems and devices [4]. Intuitive’s *da Vinci Xi* Surgical System has been marketed as offering precise dexterity, a stable video camera, improved surgeon ergonomics, and tremor elimination [5]. Robotic systems such as these have the potential to revolutionise the surgical approach to procedures, and have previously been found to increase case volume in hospitals that have invested in this new technology [6].

Despite these benefits, robotic approaches to certain procedures such as hysterectomies are associated with greater environmental impact. A recent systematic review estimated that the greenhouse gas (GHG) emissions of robotic hysterectomies was 43.5% higher and weight of waste was 24.0% higher compared to laparoscopic hysterectomies [7, 8]. Anthropogenic greenhouse gas emissions have been shown to intensify global warming, which adversely impacts the environment and leads to poorer health outcomes due to the increased risk of respiratory and infectious pathologies [9]. Thus, although RAS may contribute to improved patient recovery and overall quality of care, important considerations must be made regarding the environmental implications. The exact degree of environmental impact of RAS remains unclear, and consequently, the clinical benefits of RAS may not sufficiently outweigh the environmental cost [8].

The current literature on environmental impact of RAS is limited and a lack of quantitative studies coupled with heterogeneity of methodologies employed, impairs the ability to make objective comparisons and analyses between studies [10, 11]. Therefore, the primary aim of this study was to objectively quantify the environmental impact of RAS in a multi-specialty case load at a major public tertiary institution by determining the weight of instrument and consumable waste generated by RAS procedures.

## Methods

### Study design

This study was a retrospective analysis of prospectively collected RAS audit data at a public tertiary institution, Royal Prince Alfred Hospital (RPAH), in Sydney, Australia. The weight of waste of instruments and consumables used for each RAS case was calculated and analysed. This study was conducted under a waiver of consent approved by RPAH HREC for the BEST Database (2019/ETH08903).

### Royal prince alfred hospital surgical robotic program

The RPAH acquired Intuitive’s *da Vinci Xi* Surgical System in 2015, becoming the first public tertiary hospital in Australia to adopt this technology. The surgical robotics program at RPAH involves a comprehensive research governance framework aiming to contribute to the current body of evidence on the appropriateness of RAS usage across several specialties [12]. All patients undergoing RAS with the *da Vinci Xi* system are required to be enrolled into a prospective research database or study within RPAH. For this study, audit data was extracted from the surgical robotics program database (BEST database), which collects de-identified data from all RAS cases performed at RPAH, including instruments and consumables used. Only specialties with an annual average of more than one RAS case were included in the analysis.

### Instruments and consumables

The weight of each individual instrument and consumable in the BEST database was collected using an operating theatre digital scale (Digi DS-671). Each item on the listed was gathered from storage rooms or operating theatres and weighed, including both the item itself and the packaging it was sealed in.

All items directly associated with or specifically required for the function of the *da Vinci Xi* system were considered “robotic items” and included instrument arms, sterile drapes, seals, sheaths, covers, and stapler reloads (Online Resource 1). Any items that were either not specifically required for the operation of the *da Vinci Xi* system or not Intuitive products but were used in the procedure were classified as “other items” and included gauzes, access ports, Endocatch pouches and clips. Specialist surgeons for each specialty were consulted on which items should be categorised as robotic or otherwise. For the purposes of this study, “consumable” and “single-use” were used interchangeably, and the term “item” encompassed both instruments and consumables. Some instruments were classified as single-use while others were reusable and had multiple lives/uses, which was accounted for in all calculations (Online Resource 2).

### Number of lives

Each *da Vinci Xi* robot instrument arm has a set number of lives or uses, determined by the manufacturer, Intuitive, which decreases after each use. Once the number of lives reaches zero, the console will reject the arm, rendering it unusable, regardless of its current condition. It is noted that in certain cases instruments may require early disposal due to significant wear. To standardise the approach for this study, the assumption was made that all instrument arms were discarded only when they had zero lives left. To calculate the number of discarded robotic instrument arms, the following approach was used: A Debakey Forceps arm, with a total weight of 0.41 kg and 10 lives, was used in a procedure indicating 1 life or 10% of use has been expended. Therefore, 10% of the weight was attributed to the waste of the case (0.041 kg). In the case where two different Debakey Forceps were used, then 20.0% of the waste (0.082 kg) was attributed to the procedure. This method was applied to the calculation of all reusable robotic instrument arms. Certain robotic instrument arms have a certain number of uses before they must be discarded, for example, Clip Appliers can only be used for 100 closures. The average number of closures made per case was estimated based on available data and applied to each case.

### Weight of waste and data analysis

The weight of waste of every RAS case was calculated by multiplying the number of times each item was used by the weight of each item (in kilograms) and summating the values. Mean weight was obtained by dividing the total weight by the total number of cases for each specialty and procedure type. All statistical analyses was performed on IBM SPSS (Version 28 190). Parametric and non-parametric tests will be conducted as appropriate to determine the statistical significance of the differences in the mean annual weight of waste produced, with *p* < 0.05 defined as statistically significant. The environmental impact of RAS was evaluated by quantifying the total weight of instrument and consumable waste for each case.

## Results

**Fig. 1:**
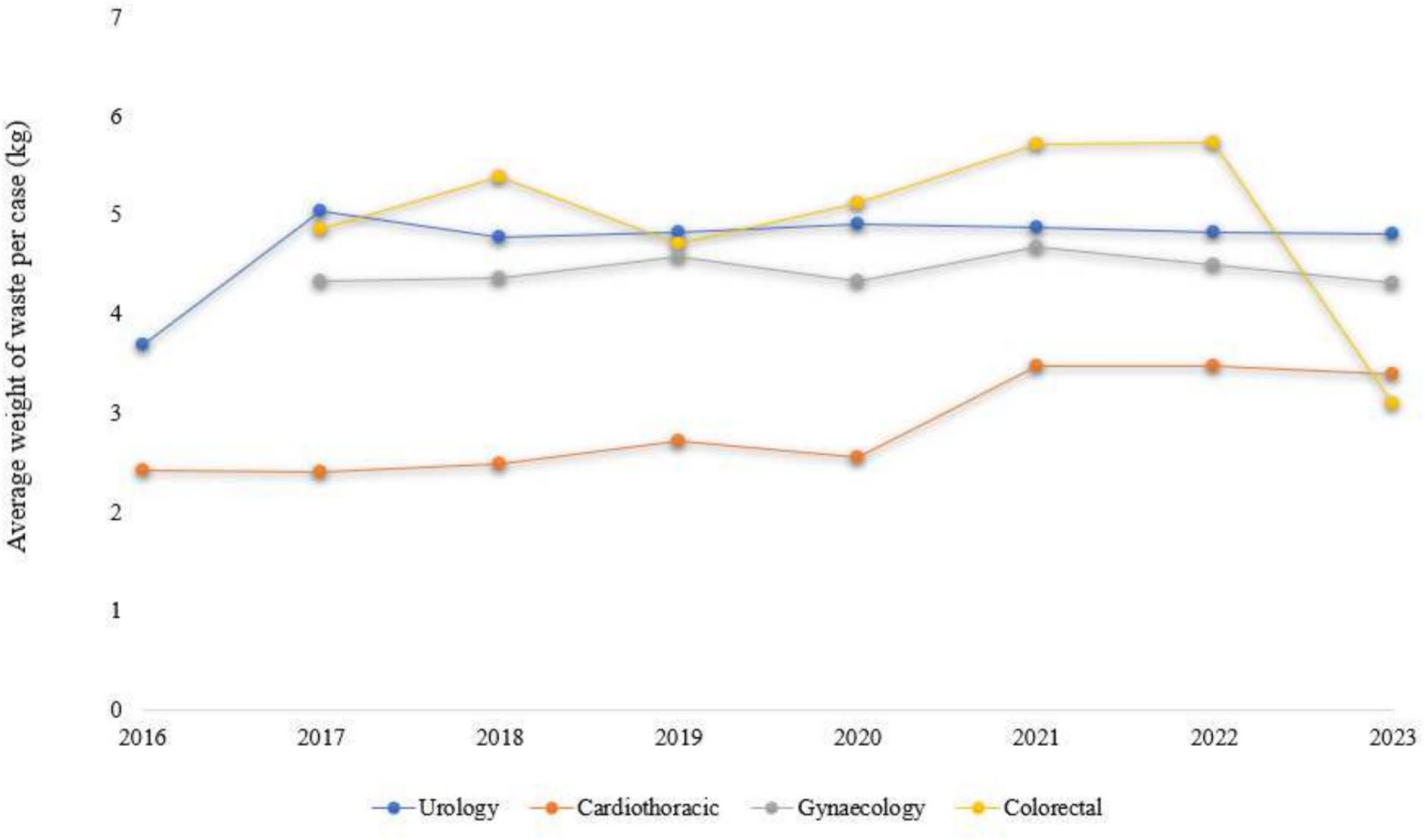
Average weight of waste per case each year

Between August 2016 and March 2023, a total of 673 patients underwent RAS using the *da Vinci Xi* System. Specialties that implemented this system included Urology (*n* = 341, 50.7%), Cardiothoracic (*n* = 158, 23.5%), Gynaecology (*n* = 107, 15.9%), Colorectal (*n* = 65, 9.6%) and Upper GI (*n* = 2, 0.2%) RAS cases, however, Upper GI had an average of less than one case per year and were subsequently excluded from annual robotic caseload. (Table 1). The monthly RAS caseload was around 8.4 cases. The total weight of discarded items over the entire study period was 2,948.90 kg. The robotic items (*da Vinci Xi* system instruments and consumables) weighed 1,364.32 kg (46.3%) while the other items that were not directly associated with the *da Vinci Xi* system weighed 1,584.62 kg (53.7%). The average weight of waste generated was 4.39 kg per case across all specialty and procedure types, over the study period (Table 2). The average weight generated per case by each specialty remained relatively unchanged throughout the years (Fig. 1).

**Table 1:** Number of patients and weight of waste of each specialty and procedure type

Procedure	No. of patients	Weight of robotic waste (kg)	Weight of other waste (kg)	Total weight of waste (kg)	Mean weight of waste per case (± SD) (kg)	Weight of robotic waste over total waste
Urology	341	624.96	1026.47	1651.43	4.84 (0.41)	37.84%
RP	310	568.12	948.41	1516.53	4.89 (0.35)	37.46%
PN	30	54.43	74.81	129.24	4.31 (0.54)	42.12%
Other^1^	1	2.41	3.25	5.66	5.66 (0.00)	42.58%
Cardiothoracic	158	361.83	117.83	479.66	3.04 (1.00)	75.43%
Lobectomy	45	141.55	42.50	184.05	4.09 (0.99)	76.91%
CABG	31	58.61	16.94	75.55	2.44 (0.32)	77.57%
Thymectomy/mass excision	30	54.07	27.13	81.20	2.71 (0.38)	66.59%
MVR	24	42.26	10.82	53.09	2.21 (0.26)	79.61%
Segmentectomy	21	53.09	16.32	69.42	3.31 (1.01)	76.49%
Other^2^	7	12.24	4.14	16.37	2.34 (0.38)	74.74%
Gynaecology	107	195.26	280.28	475.54	4.44 (0.54)	41.06%
Endometriosis excision	56	101.50	146.51	248.01	4.43 (0.63)	40.93%
Hysterectomy	39	70.61	105.56	176.17	4.52 (0.32)	40.08%
Other^3^	12	22.03	29.33	51.37	4.52 (0.41)	42.90%
Colorectal	65	182.23	160.03	342.27	5.27 (0.91)	53.24%
Colectomy	36	99.02	90.58	189.60	5.27 (1.01)	52.23%
AR	29	83.21	69.53	152.74	5.27 (0.79)	54.48%
All specialties	671	1364.29	1584.61	2948.90	4.39 (1.03)	46.26%

*RP* radical prostatectomy, *PN* partial nephrectomy, *CABG* coronary artery bypass graft, *MVR* mitral valve repair, *AR* anterior resection

^1^Other Urology procedures include: 1 Cystoprostatectomy

^2^Other Cardiothoracic procedures include: 3 Diaphragm plication, 3 Atrial septal repair, 1 Tricuspid valve repair

^3^Other Gynaecology procedures include: 4 Myomectomy, 4 Sacrocolpopexy, 1 Colpectomy, 1 Cystectomy, 1 Intrauterine device (IUD) insertion, 1 Complicated hysteroscopy and laparoscopy

**Table 2:** Annual number of patients and average weight per case

Year	No. of patients	Weight of robotic waste (kg)	Weight of other waste (kg)	Total weight of waste (kg)	Mean weight of waste per case (± SD) (kg)	*p* Value*
2016**	14	26.12	15.43	41.55	2.97 (0.71)	–
2017	80	147.36	210.45	357.81	4.47 (1.05)	< 0.001
2018	118	237.84	304.81	542.66	4.59 (0.79)	0.398
2019	131	248.93	336.31	585.24	4.47 (0.80)	0.227
2020	115	228.32	273.89	502.21	4.37 (1.14)	0.432
2021	86	200.62	179.25	379.87	4.42 (1.22)	0.766
2022	97	213.53	199.28	412.81	4.26 (1.11)	0.351
2023***	30	61.56	66.32	127.88	4.26 (0.98)	0.975

*Independent Samples t-test were performed between the average weight of waste per case for that year and the preceding year, with P values shown

**Data only available August 2016 and onwards

***Data only available up until March 2023

The weight of items were heterogenous and ranged from 0.01 kg to 1.16 kg. A total of 2,769.51 kg of single use items were disposed, contributing the majority of weight of waste (93.9%) (Online Resource 1). The *da Vinci Xi* instrument arm drape was the most used item and had the largest contribution to weight of waste in every specialty and in every procedure type (Online Resource 1). A total of 2692 instrument arm drapes were used and discarded, amounting to 969.11 kg (32.9%) of the total weight of waste (Online Resource 1). The only significant difference in mean on independent t-test for annual weight of waste was found between 2016 and 2017, which was 2.97 kg and 4.47 kg respectively (*p* < 0.001) (Table 2). However, it should be noted that the specialties of colorectal and gynaecology surgery only commenced use of the robot in 2017.

### Urology

Urology had the highest total weight of waste over the study period (1,651.43 kg) as shown in Table 1. Of those, there were 310 radical prostatectomies (4.89 kg of waste per case), 30 partial nephrectomies (4.31 kg of waste per case) and 1 cystoprostatectomy (5.66 kg). An average urology RAS case is expected to produce 4.84 kg of waste, with 37.8% being comprised of robotic items (Table 1). Monopolar scissors (*n* = 356), Large needle drivers (*n* = 337) and Prograsp forceps (*n* = 337) were the two most frequently used robotic instrument arms in urology RAS cases over the study period (Online Resource 1).

### Cardiothoracic

Cardiothoracic RAS had the lowest average weight of waste per case (3.04 kg) across eight different operative procedures. Lobectomies generated the highest average weight of waste per case among cardiothoracic procedures, at 4.09 kg, while mitral valve repairs generated the lowest at 2.21 kg (Table 1). Robotic instruments and consumables comprised a majority (75.4%) of the total weight of waste produced by cardiothoracic RAS (Table 1). Cardiothoracic RAS consistently produced the highest proportion of robotic items compared to the total waste. The number of cardiothoracic RAS cases has seen a gradual increase in recent years, from 2018 (*n* = 9) to 2022 (*n* = 41) (Table 1). The permanent cautery spatula was the most commonly used robot instrument arm by cardiothoracic, being used a total of 112 times in 158 cases (Online Resource 1). Cardiothoracic was also the specialty that most utilized the staplers (stapler 30, stapler 45, stapler 60 and their respective reloads), accounting for 13.8% of the total weight generated. The curved bipolar dissector (*n* = 43) and Debakey forceps (*n* = 25) were exclusively used by cardiothoracic, and neither were used by the other three specialties.

### Gynaecology

Most gynaecology RAS cases were either endometriosis excisions (*n* = 56) or hysterectomies (*n* = 39), each producing an average of 4.43 kg and 4.52 kg of waste respectively. The most used instrument arm by gynaecology was the monopolar hot shears scissors (*n* = 104) followed by the Maryland bipolar forceps (*n* = 91) (Online Resource 1). Endometriosis excisions and hysterectomies accounted for the majority of gynaecology RAS cases and both generated similar weights of waste, 4.43 kg, and 4.52 kg per case respectively (Table 1).

### Colorectal

Colorectal is the specialty with the lowest number of cases over the study period however, had the highest average weight of waste generated per case at 5.27 kg. Anterior resections and colectomies were the only two procedures performed robotically by colorectal surgery, and both were the procedure that was associated with the highest average weight of waste generated (5.27 kg). The tip-up fenestrated grasper was used in 47 out of 65 cases, whereas both Urology and Gynaecology rarely used this particular arm, utilising it only once each during the entire study period (Online Resource 1). The vessel sealer was also frequently in colorectal RAS cases (*n* = 57 in 65 cases) which accounted for approximately 10.3% of the total waste generated by the specialty. The other three specialties used the vessel sealer for a combined total of four times.

## Discussion

### Statement of principal findings

This study objectively quantified the environmental impact of RAS at a major public tertiary institution and is, to the authors’ knowledge, the first study in the literature to investigate the material waste of a multidisciplinary RAS case load in Australia. Across all specialties, the average weight of waste produced by a RAS case is approximately 4.39 kg.

During the 2021/2022 financial year, there was an estimated 18,208 RAS cases performed using the *da Vinci Xi* system in Australia, and extrapolating the results of this study leads to an estimated 79,934 kg of waste [13, 14]. According to Intuitive’s Q1 Investor Report, it is projected that during the 2031/2032 financial year in Australia (assuming the purported 18% year-on-year growth rate) 78,765 RAS cases will be performed [15], equating to 345,778 kg of waste produced.

### Strengths and weaknesses of the study

This study’s design was largely based on previous studies in the literature [16, 17]. Both studies measured the weight of waste generated (kilograms) by certain procedures, Thiel et al. [16] prospectively investigated phacoemulsification surgeries at two tertiary centres, while Woods et al. [17] retrospectively investigated three different surgical modalities: the laparotomy, conventional laparoscopy, and robotic-assisted laparoscopy. Both previous studies supported the scientific validity of the methodology used in the current study but only focused on a single procedure type within one specialty. In contrast, this study expanded the scope to include a multi-specialty case load, which enabled objective comparisons between specialties and procedures. Comparative assessment of surgical approaches is limited by a lack of prospectively collected data on instrument usage for equivalent laparoscopic and open approaches to assess the difference in waste generation between surgical approaches.

The methodology for measuring the environmental impact of RAS varies significantly in the current literature, contributing to the discrepancies in results and findings. A common methodology involves a LCA, which is an internationally standardised method used to investigate, estimate, and evaluate the environmental burden caused by a product, process, or service throughout its lifespan [18]. Woods et al. [17] utilised the weight of waste generated (kg) and amount of energy consumed (kWh) per RAS case as LCA inputs and found that 40.3 kg of CO2 equivalents (CO2e) was associated with each robotic-assisted laparoscopy. While Unger et al. [19] performed a LCA analysis on the material composition of the waste yielded by RAS and calculated the environmental impact of substituting biopolymers over petroleum-based plastics for medical item production. Alternate methodologies include the cost of opened but unused operating theatre instruments [20] or the lifetime of robotic arms [21].

### Strengths and weaknesses in relation to other studies

This study found that single-use instruments and consumables contributed the majority of the total weight of waste of RAS (93.9%). Woods et al. [17] conducted a similar study investigating the environmental impact of three different approaches to endometrial cancer staging (laparotomy, laparoscopic and robotic) and found that up to 65.5% of the total waste associated with the RAS approach was also due to single-use devices and consumables. The difference in the procedure performed may account for the large disparity. The instrument arm drape, made of polyethylene and used to prevent contamination of the robotic instrument arms, was the item with the greatest contribution to the total waste generated across all specialties and procedures (32.9%). A US study conducted by Thiel et al. [7] also found that this type of material contributed a large portion of robotic-assisted hysterectomies’ waste stream by weight. In urology, the most used robotic instrument arm was the monopolar scissors, which needed exchanging in 10.4% of cases. This was aligned with a U.S study by Ludwig et al. [21], who reported that during robot-assisted radical prostatectomy, the monopolar scissors needed to be exchanged in approximately 12.4% of cases, with similar rates found for robot-assisted partial nephrectomies. Different countries may utilise similar instruments when performing a similar RAS procedure.

In gynaecology, Thiel et al. [7] reported that 13.7 kg of waste was generated by each robotic-assisted hysterectomy, much higher than the observed average of only 4.52 kg in the current study. This may be attributed to the difference in method of data collection as they collected waste bins at the end of procedures and analysed its contents. Consequently, other plastics items were included in their calculations, including for example surgical gowns and gloves, which were not recorded within the institutional database.

### Meaning of the study

The environmental impact of surgical waste must be considered within the broader context of global healthcare waste generation. Comparisons to other industries offer insight into the magnitude of this global issue. The healthcare industry is the second largest global waste generator, surpassed only by the food industry, yet more than both the automotive and energy industries [24]. The United States healthcare system produces roughly 1.8 billion kilograms of waste annually, the most out of any country globally [24] and it is estimated that up to 70% originated from the operating theatre [33]. Australia has a significant role to play in tackling this issue because it possesses the second largest healthcare-related carbon footprint per capita in the world, second only to the US, generating 1.29 tonnes of CO2e per capita [35]. In 2014–2015, the carbon footprint attributed to the healthcare system in Australia was 35,772 of 494,930 kilotonnes of CO2e, 7% of Australia’s total; with the majority generated by hospitals (44%) [36]. These figures are likely much higher today. To reduce the national and global carbon footprint, integration of more carbon-efficient practices, for healthcare workers, institutions, and companies, is essential, with some of the aforementioned interventions being possible solutions.

Few studies have evaluated waste generation across different surgical approaches to assess the environmental impact of RAS compared to equivalent laparoscopic and open approaches. A comparative analysis of hysterectomy procedures demonstrated that robotic-assisted hysterectomies produced up to 30% more material waste by weight than laparoscopic and open approaches, contributed to mostly by plastic, paper and cardboard packaging [7]. Comparatively, a study assessing the environmental impact of radical prostatectomy demonstrated comparable weight of instrument waste produced in RAS and an 11% lower carbon footprint due to lower operative time and lower CO2 emissions when compared to laparoscopic surgery for equivalent cases [34]. These findings highlight the need for targeted strategies to minimise waste across surgical modalities while considering their clinical benefits.

In this study, waste was quantified by weight, however disposal complexity is an important consideration due to the material composition and environmental challenges associated with operating theatre waste which is crucial for determining associated impact and cost. General waste is sent to landfills and comprises of non-hazardous, uncontaminated materials such as equipment packaging. The volume of single use plastics in packaging of consumables generates a considerable amount of general waste associated with RAS. Hazardous waste, however, poses more significant health risks and require specialized disposal processes. These include sharps, pharmaceuticals, and equipment contaminated by bodily substances. Roughly 60% of hazardous waste is incinerated while the remainder is re-sterilized at the sterilization department of each hospital. The largest contributors to waste generated during RAS including the instrument arm drapes contribute substantially to hazardous operating theatre waste. Given the increased complexity, processing of hazardous waste costs on average 10–20 times more than that of general waste [25]. The by-products of these processes are also known to be associated with adverse environmental and health impacts [26, 27].

Single-use disposables contributed the majority of the weight of waste in this study. It has been shown that single-use medical items cost more, produce more CO_2_ and consume more water than their reusable counterparts [22]. The healthcare industry globally has seen a systemic rise in usage of single-use consumables over the years due to the advantages of enhanced sterility and the reduced risk of contamination [23]. Since 1992, waste production in healthcare has been increasing by at least 15% annually, driven in part by the growing reliance on disposable items [24].

The recycling of single-use consumables or increasing adoption of reusable items should be advocated as a possible intervention to minimise waste generation Additionally, proper waste segregation by theatre staff has also been shown to maximise recyclability of waste generated from operating theatres [28]. A local study examined the benefits of waste segregation and recycling at a public tertiary institution in Queensland, Australia [29]. They found that through careful waste segregation into general, clinical, and recyclable waste, the institution saw a reduction in waste management costs by approximately $10,200AUD per month, and increasing staff education should be considered to improve waste segregation behaviour and to potentially obtain similar results [30].

Remanufacturing of single-use medical instruments has been shown to be an effective method of reusing certain medical equipment, and may potentially be an effective intervention [31] and may even assist in combating the poor availability of medical items in developing countries [32]. In the institution, this study was conducted in, it was estimated an approximate $107,680AUD could be saved through decreasing 16.4% the total weight of waste over the study period through reusing the drapes.

### Unanswered questions and future research

The current literature on the topic of environmental impact of RAS is limited, with most studies investigating a single procedure within a single specialty. Further research should seek to compare waste production between surgical approach (robotic-assisted, laparoscopic and open) for equivalent procedures using prospectively collected individual case data to further evidence on the environmental impact of RAS. Moreover, environmental impact should be heightened as a consideration when implementing and supporting surgical robotics programs with effective environmental solutions proposed and considered by administrators and healthcare professionals performing RAS. Institutions should seek opportunities to consider the environmental impact of RAS and, where appropriate assess unnecessary and instead advocate for non-robotic alternatives as they create reduced ecological impact [7]. Although most procedures confer additional clinical benefit when performed robotically, this is not the case for all procedures. Recent systematic reviews found that robotic approach to cholecystectomy and rectal cancer excision was not superior to laparoscopic approach [37, 38]. Notably, reducing the weight of waste produced by certain procedures are not associated with poorer health outcomes [39]. Understanding these aspects can lead to more environmentally conscious decisions in surgical practices, ensuring a more sustainable future for RAS and a more positive effect on the planet, without compromising patient outcomes.

## Conclusions

This study objectively quantified the environmental impact of RAS at a major public tertiary institution and identified key opportunities for targeted interventions to improve sustainability. Although RAS offers numerous clinical benefits, the associated environmental impact cannot be ignored and should be incorporated into clinical decision making. It is imperative that healthcare administrators and providers consider sustainability efforts especially in the uptake of robotic surgery. A collaborative effort between healthcare professionals, institutions and companies can help create a more sustainable and environmentally friendly future for robotic surgery. This study hopes to assist with decision-making surrounding the implementation and use of RAS and help form a foundation for future research to develop more effective and efficient interventions to reduce its environmental impact.

## Supplementary Information

Below is the link to the electronic supplementary material.

Supplementary file1 (XLSX 21 KB)

Supplementary file2 (PDF 84 KB)

## Data Availability

No datasets were generated or analysed during the current study.
