# Supplementary material for: The environmental impact of multi-specialty robotic-assisted surgery: a waste audit analysis
Source: J Robot Surg. 2025 Mar 11;19(1):113. doi: 10.1007/s11701-025-02278-5 (PMC11897078; doi:10.1007/s11701-025-02278-5)

Journal of Robotic Surgery

THE ENVIRONMENTAL IMPACT OF MULTI-SPECIALTY ROBOTIC-ASSISTED SURGERY: A WASTE AUDIT ANALYSIS

Gerald Tjahyadi[a,b], Patrick-Julien Treacy[a,c], Kate Alexander[a,c,d], Jacob Bird[a,c,d], Sascha Karunaratne[a,c,d], Scott Leslie[c,d,e], Kate McBride[a,c], Daniel Steffens[a,c,d], Ruban Thanigasalam[c,d,f]

[a] Surgical Outcomes Research Centre (SOuRCe), Royal Prince Alfred Hospital, Sydney, NSW, Australia

[b] Faculty of Medicine and Health, Concord Clinical School, The University of Sydney, Sydney, NSW, Australia

[c] Institute of Academic Surgery (IAS), Royal Prince Alfred Hospital, Sydney, NSW, Australia

[d] Faculty of Medicine and Health, Central Clinical School, The University of Sydney, Sydney, NSW, Australia

[e] Department of Urology, Royal Prince Alfred Hospital, Sydney, NSW, Australia

[f] Department of Urology, Concord Repatriation General Hospital, Sydney, NSW, Australia

Corresponding author: Gerald Tjahyadi
Surgical Outcomes Research Center (SOuRCe)
Royal Prince Alfred Hospital
PO Box M157 Camperdown, Sydney NSW 2050, Australia.
E-mail: gtja2217@uni.sydney.edu.au

Online Resource 2. Diagram of item classification with a non-exhausting list of examples

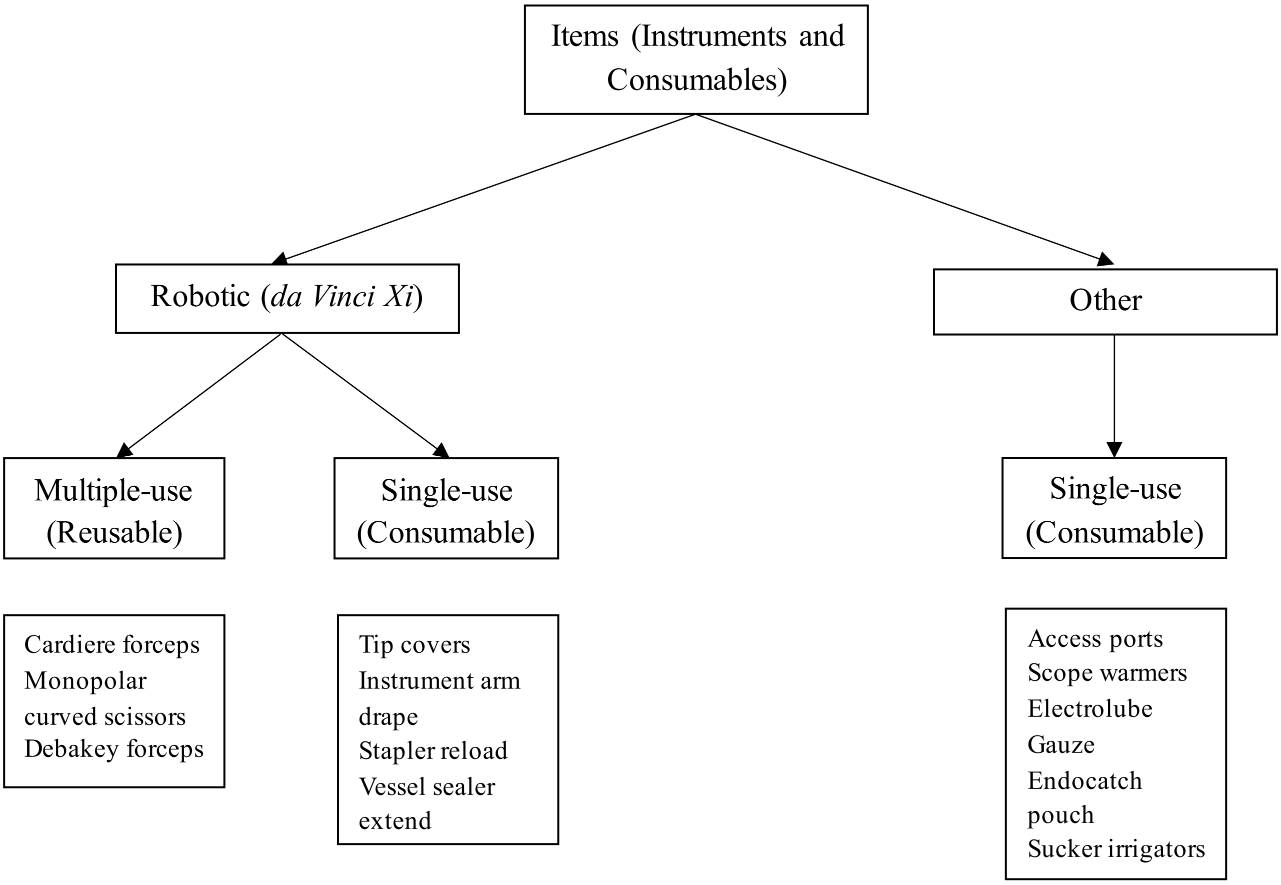

Supplement: Supplementary file 2 — Supplementary file2 (PDF 84 KB) [file 11701_2025_2278_MOESM2_ESM.pdf]
